# Long-Term Outcomes of Femorofemoral Crossover Bypass Versus Endovascular Revascularization in Iliac Artery Occlusions: A Retrospective Study

**DOI:** 10.3390/jcm14062109

**Published:** 2025-03-19

**Authors:** Edin Ahmic, Paul Swatek, Iurii Mykoliuk, Anton Busau, Muhammed Abdallah, Wolfgang Hitzl, Klaus Linni, Ara Ugurluoglu, Jörg Lindenmann

**Affiliations:** 1 Division of Thoracic and Hyperbaric Surgery, Department of Surgery, Medical University of Graz, 8036 Graz, Austria; 2 Department of Cardiovascular and Endovascular Surgery, Paracelsus Medical University Salzburg, Müllner Hauptstrasse 48, 5020 Salzburg, Austria; 3 Research Office (Biostatistics), Paracelsus Medical University, Müllner Hauptstrasse 48, 5020 Salzburg, Austria

**Keywords:** femorofemoral, bypass, artery occlusions, perioperative

## Abstract

**Objective**: The objective of this study is to compare the long-term outcomes of femorofemoral crossover bypass (FCOB) and endovascular treatment (ET) in managing iliac artery occlusions. **Methods**: The data of 200 patients with iliac artery lesions who were treated at a single center within 7 years were evaluated retrospectively. Of these, 82 (41%) underwent FCOB, and 118 (59%) received ET. Primary outcomes included patency, limb salvage, and survival rates, while secondary outcomes assessed complications, including wound infections and restenosis. Follow-up was conducted over a median of 4.98 years. **Results**: Primary patency (PP) rates after 3 years were 80% for FCOB and 88% for ET. Primary assisted patency (PAP) was 95% for FCOB and 93% for ET. Secondary patency (SP) was 97% for FCOB and 98% for ET. Both FCOB and ET achieved comparable long-term outcomes in limb salvage, 94% in both groups at 8 years. ET demonstrated advantages in shorter hospital stays (1.49 ± 2.51 vs. 8.21 ± 9.82 days, *p* < 0.0001) and lower perioperative complications, including transfusion rates (3.4% vs. 13.4%, *p* = 0.01226). However, FCOB exhibited lower restenosis rates (6.1% vs. 20.39%, *p* = 0.00441), despite a higher rate of reocclusion (19.5% vs. 6.8%, *p* = 0.00800). Survival rates at 8 years were 54% for FCOB and 67% for ET. **Conclusions**: ET is the preferred first-line approach due to its minimally invasive technique, shorter recovery time, and fewer complications. FCOB remains essential for patients with complex lesions or when ET is not feasible, offering durable long-term outcomes. Appropriate treatment selection should consider both the patient’s condition and clinical and anatomical factors to optimize the best possible patient outcomes.

## 1. Introduction

Peripheral artery disease (PAD) affecting the iliac arteries can present as intermittent claudication or progress to chronic limb-threatening ischemia (CLTI) in advanced cases. These conditions arise from significant arterial occlusions, which lead to reduced blood flow to the lower limbs, manifesting as ischemia, pain, tissue loss, or functional limitations [1,2,3]. Revascularization, whether through open surgical or endovascular techniques, is a pivotal intervention for restoring perfusion, alleviating symptoms, and preventing complications in affected patients [3].

Two primary revascularization strategies are frequently utilized for iliac artery occlusions: femorofemoral crossover bypass (FCOB) [4] and endovascular treatment (ET) [5,6]. FCOB was first described by Freeman and Leeds in 1952 as an effective surgical approach for managing patients with unilateral iliac occlusive disease [7]. Originally developed as an alternative to anatomic reconstruction, FCOB has since become a reliable option. Although ET has become more commonly applied for iliac stenosis and occlusion [8], FCOB remains essential, particularly for extensive iliac occlusions where endovascular treatments may not provide sustainable results [9].

The Trans-Atlantic Inter-Society Consensus II (TASC II) guidelines generally do not recommend FCOB bypass as the first-choice surgical technique for revascularization. Nevertheless, in recent years, endovascular therapy has been favored as the first-line treatment for peripheral arterial disease involving iliac lesions due to its minimally invasive approach and reduced perioperative risks. While surgical revascularization continues to be the standard for TASC II type D iliac lesions, ET has demonstrated promising results even in complex cases. However, anatomical complexities such as long-segment occlusions or heavily calcified lesions often necessitate surgical approaches like FCOB. Advances in surgical techniques and instrumentation have further broadened the indications for FCOB, establishing it as a viable option not only for high-risk patients but also for those with moderate-risk profiles or significant symptoms of claudication [10].

Although both FCOB and ET are widely utilized, direct head-to-head comparisons of their long-term outcomes remain exceedingly rare, with only a limited number of studies addressing this topic comprehensively [11]. While numerous studies have reported outcomes for FCOB [9,12,13,14] and ET [6,8,15] separately, there is a notable lack of research directly comparing these techniques in terms of limb salvage, patency rates, and survival outcomes. This gap in the literature emphasizes the need for further investigation to determine which method offers superior long-term benefits, particularly for patients with complex iliac occlusions.

FCOB serves as an effective alternative to aorto-unifemoral bypass for patients with iliac artery occlusion and chronic limb-threatening ischemia, especially in individuals with compromised general health [12,16,17]. In this study, we aim to address this gap by retrospectively analyzing and comparing the long-term outcomes of FCOB and ET for iliac artery occlusions. This retrospective study seeks to evaluate and compare the outcomes of FCOB and ET in terms of limb salvage, patency rates, and overall survival in patients with iliac artery occlusions.

## 2. Materials and Methods

### 2.1. Study Design

This retrospective study analyzed data from patients with iliac artery occlusions treated with either FCOB or ET between 1/2014 and 12/2020 at the University Hospital Salzburg, Austria. All patients who underwent endovascular treatment in this study received stenting as part of the procedure. Furthermore, the study was approved by the local ethics committee (Nr. 1143/2023). The study population included individuals with advanced iliac artery occlusive disease, classified under TASC II and the Global Limb Anatomic Staging System (GLASS). These classifications represent complex, long-segment occlusions often associated with chronic limb-threatening ischemia (CLTI).

All patients included in the study had intermittent claudication or severe ischemia symptoms, such as rest pain or tissue loss (Rutherford categories 4–6). They were evaluated for suitability for either surgical or endovascular revascularization based on clinical and anatomical factors. The choice between FCOB and ET was made individually for each patient based on clinical and anatomical factors. All patients were assessed based on predefined anatomical and clinical criteria, including TASC II classification, GLASS staging, and symptom severity. The decision between FCOB and ET was primarily based on the visual assessment of CT angiography (CTA) and duplex ultrasound (DUS), particularly the extent of calcifications and lesion characteristics. Patients with extensive calcifications and long-segment occlusions were more likely to undergo FCOB, while those with shorter, less calcified lesions typically received ET. Baseline characteristics were comparable between groups, reducing the risk of selection bias. However, due to variability in surgical judgment, the final decision was made on a case-by-case basis. Inclusion criteria were as follows: patients with unilateral iliac artery disease presenting with lifestyle-limiting claudication or CLTI, suitable anatomy for intervention (TASC II type C/D lesions and patent distal outflow), and age 18 years or older. Exclusion criteria were acute iliac artery occlusion, prior ipsilateral FCOB or aortoiliac bypass, previous ipsilateral ET within the last 12 months, and pregnancy.

### 2.2. Vascular Lesion Anatomy 

The vascular lesion was categorized by the type of iliac artery involvement (common iliac artery, external iliac artery, or both), lesion type (stenosis or occlusion), and lesion length. The mean lesion length was recorded for both groups. Additionally, the number of patent runoff vessels was documented, and lesions were classified according to the TASC II guidelines for iliac artery disease. Preoperative assessment included clinical examinations, DUS, CTA, and/or magnetic resonance angiography (MRA).

### 2.3. Surgical Technique

The procedure was performed under general, local, or regional anesthesia, depending on the patient’s condition. Bilateral femoral vascular regions were exposed using standard longitudinal groin incisions. The common, superficial, and deep femoral arteries were dissected and controlled. A suprapubic prefascial subcutaneous tunnel was created to connect the two femoral regions, with blunt finger dissection used to minimize the risk of injury to the abdominal cavity or bladder. Dacron (Vascutek, Inchinnan, Scotland, UK) and ring-reinforced PTFE (Gore, Flagstaff, AZ, USA) grafts were used as graft materials. These grafts were passed through the tunnel and shaped in a gentle curve to prevent tension or kinking at the anastomotic sites.

After systemic heparinization, the common, superficial, and deep femoral arteries were clamped and incised longitudinally. The graft was first connected to the donor artery and then to the recipient artery to minimize blood loss and occlusion time. End-to-side anastomoses were sewn using 5-0 Prolene sutures to ensure precision and durability. If atherosclerotic plaques were present on the anterior wall of the femoral artery, or, if the artery was heavily calcified, an endarterectomy was performed to facilitate the anastomosis. These cases required a more extensive surgical approach to ensure turbulence-free blood flow into the outflow vessels. For endovascular treatments, standard techniques were applied, including subintimal recanalization and plain balloon angioplasty, using stents such as Pulsar-18 (Biotronik, Berlin, Germany) or Periflow (Bentley, Hechingen, Germany). Technical success was defined as the successful restoration of blood flow through the treated segment, confirmed intraoperatively via angiography or duplex ultrasound.

### 2.4. Follow-Up

Patients underwent either crossover bypass surgery or endovascular treatment. The follow-up investigation which was identical for both treatment arms were at 1, 3, 6, and 12 months, and then annually. Duplex ultrasound was used during follow-ups to monitor arterial patency, while clinical evaluations focused on limb salvage and symptom improvement. For patients undergoing FCOB surgery, lifelong antiplatelet therapy with 100 mg of acetylsalicylic acid or 75 mg of clopidogrel was prescribed, except in cases requiring oral anticoagulation. After ET, 75 mg of clopidogrel was added for six weeks alongside preoperative medication.

Primary endpoints included primary and secondary patency, as well as limb salvage. Secondary endpoints were primary assisted patency, target lesion revascularization (TLR), and survival. Limb salvage was defined as avoiding major amputation (above the ankle). Major amputation was defined as any amputation above the ankle, including transfemoral and transtibial amputations. Minor amputation referred to distal limb amputations, such as toe or forefoot amputation, without affecting ambulatory function. Primary patency indicated that the vessel remained open and maintained blood flow without additional intervention. Secondary patency referred to restoring blood flow through reintervention after occlusion. Primary assisted patency included minor interventions to maintain consistent blood flow. The reintervention rates were recorded for target lesion revascularization (TLR) and target extremity revascularization (TER). TLR referred to procedures performed at the initial site of revascularization—either at the bypass graft anastomoses in the FCOB group or at the iliac stented segment in the ET group. In the FCOB group, interventions included thrombectomy, patch angioplasty, or revision of the graft, while, in the ET group, repeat angioplasty, with or without additional stenting, was the predominant approach. TER included additional procedures addressing progression of disease in femoropopliteal or infrapopliteal arteries. These interventions varied between endovascular and open surgical approaches

Hemodynamic improvement was defined as an increase in the ankle-brachial index (ABI) by ≥0.10 or achieving an ABI of ≥0.9.

### 2.5. Statistical Analysis

Data were checked for consistency and normality. Fisher’s Exact test or Pearson’s test were used to analyze cross-tabulations. Generalized linear models based on lognormal distributions were used, if continuous variables deviated from normality and followed lognormal distributions.

Competing risk models with death as competing risk were used to compare primary, primary assisted, secondary patencies, and limb salvage. Kaplan–Meier analysis was used to compare overall survival.

All reported tests were two-sided, and *p*-values < 0.05 were considered statistically significant. All statistical analyses in this report were performed by use of NCSS (NCSS 2022, NCSS, LLC., Kaysville, UT, USA), STATISTICA 13 (Hill, T. & Lewicki, P. Statistics: Methods and Applications. StatSoft, Tulsa, OK, USA), and PASW 29 (IBM SPSS Statistics for Windows, Version 29.0., Armonk, NY, USA).

## 3. Results

A total of 200 patients who underwent surgery between 1/2014 and the 12/2020 were included in this study. FCOB was performed in 82 patients, while ET was performed in the remaining 118 patients. The clinical characteristics of the patients are given in Table 1. Significantly more patients in the bypass group had previously undergone coronary artery bypass grafting, with 13 patients (15.9%) compared to 6 patients (5.1%) in the ET group (*p* = 0.01). Furthermore, there was also a significant difference in the number of current smokers, with 35 patients (42.7%) in the FCOB group compared to 80 patients (68.3%) in the ET group (*p* = 0.0005). Patients with cerebral arterial occlusive disease (CAOD) were similarly distributed between groups (35.4% vs. 38.1%, *p* = 0.76). All other characteristics were equally distributed between the three groups. There was no significant difference between the groups regarding the TASC II classifications for aortoiliac occlusive disease, GLASS classifications for femoropopliteal segments, or runoff quality of infrapopliteal arteries shown in Table 2. Both the preoperative and postoperative Rutherford categories were comparable between the FCOB and ET groups, with no significant differences observed. The distribution of patients with claudication and those with CLI was balanced across both groups, indicating a similar baseline level of disease severity. Technical success was achieved in 100% in both groups.

The median follow-up was 4.98 years (IQR: 3.36–6.92 years), with a maximum follow-up duration of 8 years. The FCOB and ET groups showed similar outcomes across multiple parameters. There were no significant differences regarding the affected artery common iliac artery (CIA), external iliac artery (EIA), or both, stenosis or occlusion patterns, or stenosis lengths (mean length: 53.1 mm for FCOB vs. 47.3 mm for ET, *p* = 0.183). Likewise, reference vessel diameters for the CIA (mean diameter: 9.63 mm for FCOB vs. 9.27 mm for ET, *p* = 0.136) and EIA (mean diameter: 8.07 mm for FCOB vs. 7.81 mm for ET, *p* = 0.095) were comparable. Pre-intervention ABI values were 0.45 in the FCOB group and 0.51 in the ET group (*p* = 0.15), and post-intervention ABI values were 0.77 and 0.81, respectively (*p* = 0.40). These results highlight the overall similarity in outcomes between the two groups.

**Figure 1:**
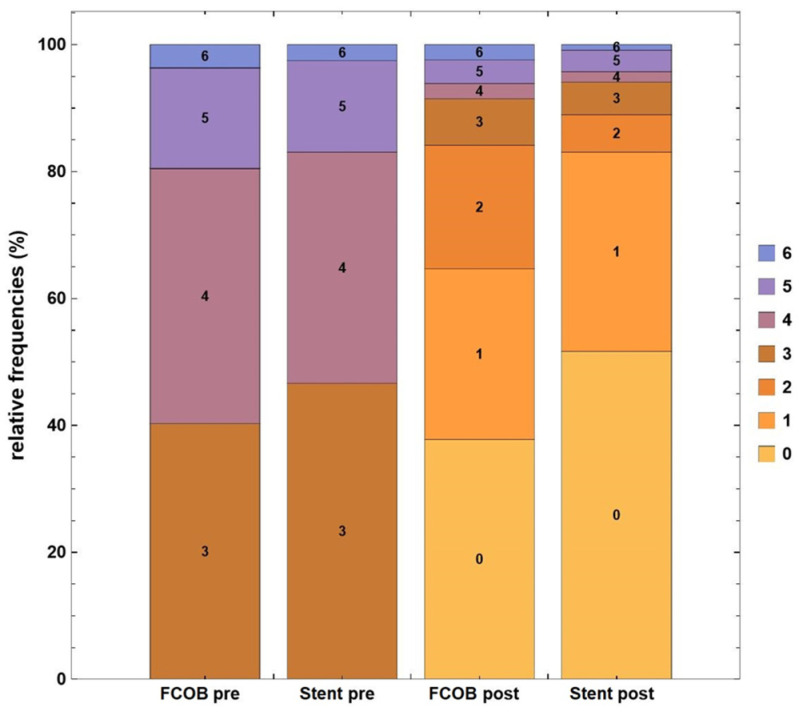
Rutherford categories before and after revascularization. Stacked bar chart illustrating the distribution of Rutherford categories in patients undergoing femorofemoral crossover bypass (FCOB) or endovascular treatment with stent. *p* = 0.432.

In the FCOB group, patients showed significant improvement in Rutherford categories following revascularization. Prior to surgery, 40% of patients were in Rutherford category 4, and 18% were in categories 5 or 6, indicating severe ischemia. Postoperatively, 90% of patients achieved Rutherford categories 0–2, reflecting substantial clinical improvement, while only 10% remained in category 3 or higher. The mean improvement in Rutherford category was 2.7 ± 1.3 (*p* < 0.001). Similarly, the ET group demonstrated a marked improvement. Preoperatively, 38% of patients were classified as Rutherford category 4, and 11% were in categories 5 or 6. After intervention, 87% of patients reached Rutherford categories 0–2, while 13% remained in category 3 or higher. The mean improvement in Rutherford category in the ET group was 2.5 ± 1.2 (*p* = 0.001). When comparing the two groups, the proportion of patients achieving Rutherford categories 0–2 postoperatively was slightly higher in the FCOB (90%) compared to the ET (87%), though this difference was not statistically significant (*p* = 0.432). Both groups achieved excellent outcomes in terms of clinical improvement. This improvement is clearly illustrated in Figure 1.

**Figure 2:**
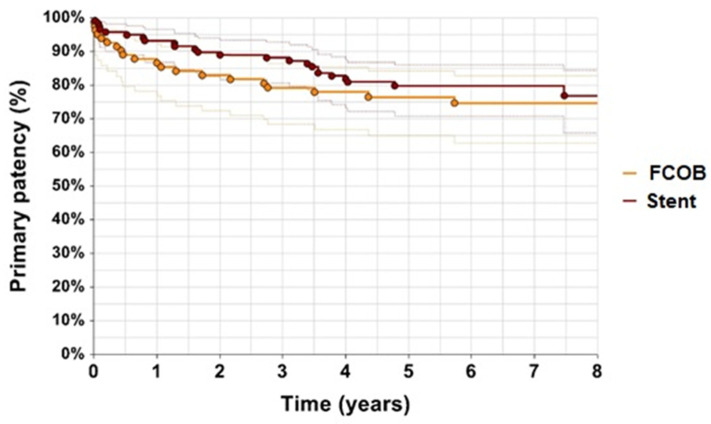
Primary patency. Kaplan–Meier estimates of primary patency with confidence intervals, comparing patients undergoing femorofemoral crossover bypass FCOB or endovascular treatment with stent. *p* = 0.29.

**Figure 3:**
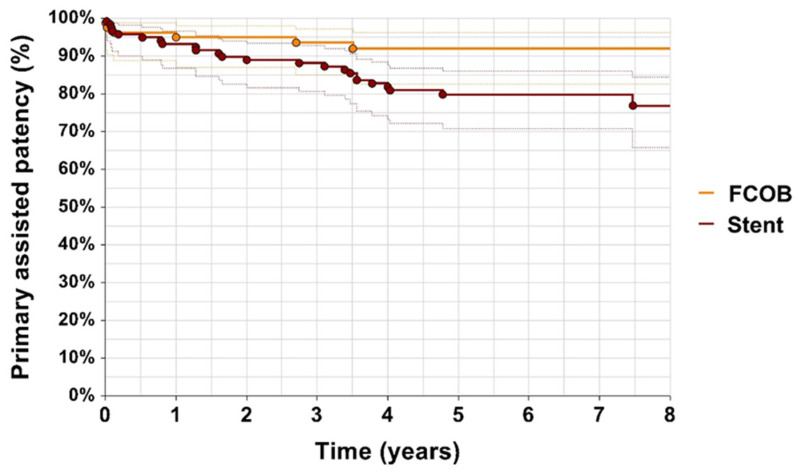
Primary assisted patency. Kaplan–Meier estimates of primary assisted patency with confidence intervals, comparing patients undergoing femorofemoral crossover bypass (FCOB) or endovascular treatment with stent. *p* = 0.066.

Primary patency rates were higher in the FCOB group compared to the ET group (Figure 2). After 1 year, 88% of patients in the FCOB group maintained primary patency, whereas 95% of patients in the ET group had primary patency (*p* = 0.29). By year 3, the primary patency rate for FCOB was 80%, while, in the ET group, it dropped to 88%. Primary assisted patency rates also favored the FCOB group. After 1 year, primary assisted patency was 95% in the FCOB group and 93% in the ET group. By year 3, in the FCOB group, it was 93%, compared to 90% in the ET group (*p* = 0.066) illustrated in Figure 3. While the differences in primary assisted patency approached statistical significance, they suggest that FCOB patients required fewer reinterventions to maintain adequate arterial flow compared to ET patients.

**Figure 4:**
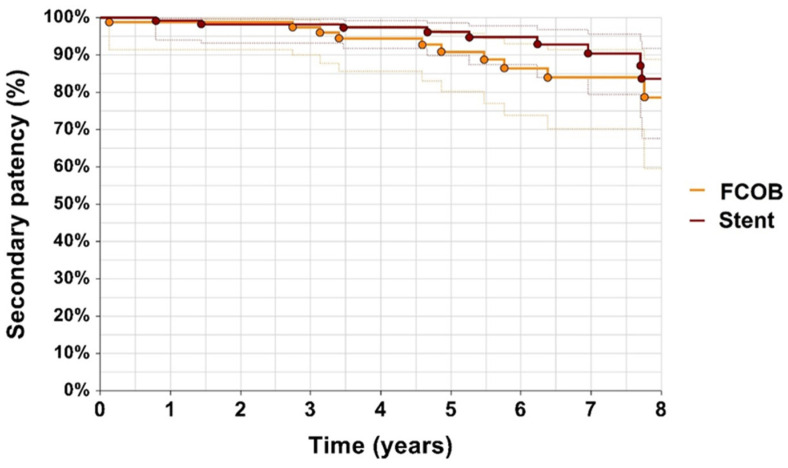
Secondary patency. Kaplan–Meier estimates of secondary patency with confidence intervals, comparing patients undergoing femorofemoral crossover bypass (FCOB) or endovascular treatment with stent. *p* = 0.28.

Secondary patency was high in both groups throughout the follow-up period. At year 1, the secondary patency rate was 98% for FCOB compared to 99% for ET. By year 3, FCOB maintained a 97% secondary patency rate, while ET dropped to 98% (*p* = 0.28) as shown in Figure 4.

**Figure 5:**
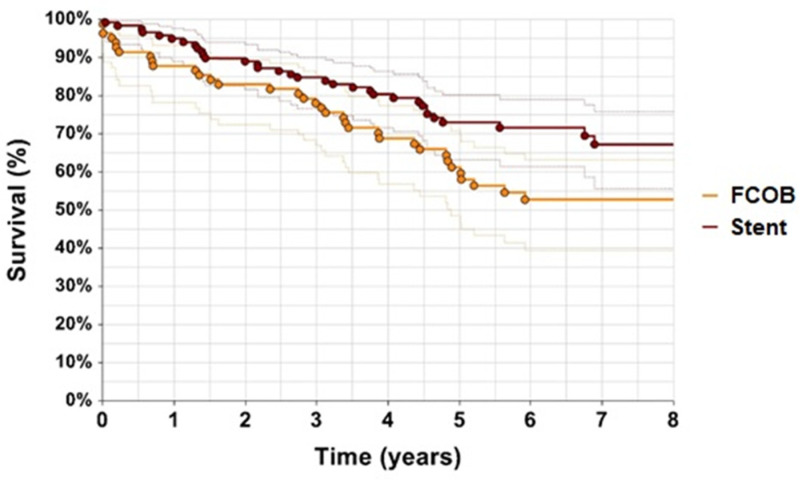
Cumulative survival. Kaplan–Meier estimates of cumulative survival with confidence intervals, comparing patients undergoing femorofemoral crossover bypass (FCOB) or endovascular treatment with stent. *p* = 0.039.

By year 8, the survival rates were approximately 54% for the FCOB group and 67% for the ET group. While the ET group demonstrated slightly higher survival rates in the earlier years, particularly within the first year (95% vs. 88%, *p* = 0.039), the long-term follow-up revealed comparable survival outcomes between the two groups. The survival rates for FCOB were 88% at 1 year, 60% at 5 years, and 55% at 8 years, while the ET group demonstrated 95% at 1 year, 73% at 5 years, and 67% at 8 years. The survival rates for ET were consistently higher across all time points, with a statistically significant difference (*p* = 0.039), as showed in Figure 5.

**Figure 6:**
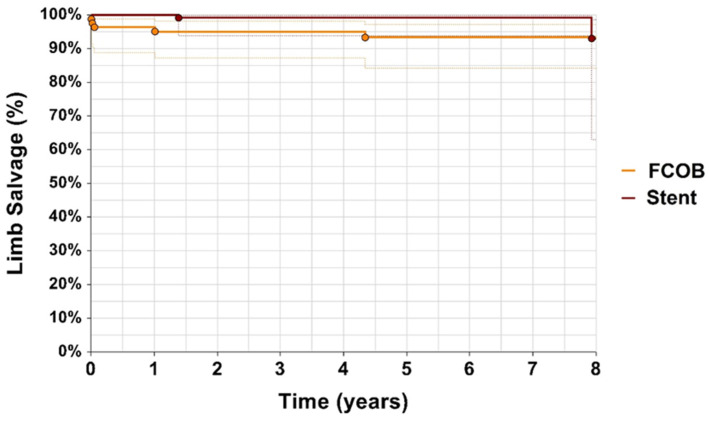
Limb salvage. Kaplan–Meier estimates of limb salvage with confidence intervals, comparing patients undergoing femorofemoral crossover bypass (FCOB) or endovascular treatment with stent. *p* = 0.064.

Limb salvage rates were high in both groups, with no significant difference observed (*p* = 0.064). After 1 year, the limb salvage rate was 96% for FCOB and 100% for ET. After 5 years, limb salvage was 95% in the FCOB group compared to 98% in the ET group, and, at 8 years, the rates were 94% for both groups illustrated in Figure 6. Both treatment approaches demonstrated comparable long-term limb preservation.

The postoperative hospital stay was significantly longer in the FCOB group compared to the ET group, with a mean of 8.21 ± 9.82 days versus 1.49 ± 2.51 days, respectively (*p* < 0.0001). Major amputations were observed in five patients (6.1%) in the FCOB group and two patients (1.7%) in the ET group, while minor amputations occurred in three patients (3.7%) in the FCOB group and two patients (1.7%) in the ET group. Neither difference reached statistical significance (major amputation: *p* = 0.13; minor amputation: *p* = 0.40).

Distal embolization occurred in 5 patients (6.1%) in the FCOB group, and blood transfusions were required in 11 patients (13.4%) in the FCOB group compared to 4 patients (3.4%) in the ET group, showing a statistically significant difference (*p* = 0.01226). Bleeding or pseudoaneurysms were observed in 5 FCOB patients (6.1%) and 7 ET patients (5.9%), with no significant difference between the groups (*p* = 1.0) as illustrated in Table 3.

Wound infections were observed exclusively in the FCOB group, affecting 19 patients (23.2%). Among these, 12 cases were superficial surgical site infections (SSIs), which were successfully managed with negative pressure wound therapy. Seven cases were deep SSIs, requiring surgical revision. Of the deep infections, four involved grafts that needed removal and replacement. Two of the replacement grafts used were autologous vein grafts from the vena saphena magna, while the other two were synthetic Dacron^®^ grafts. In the majority of cases, Dacron grafts were used in 67 patients (82%), while 15 patients (18%) received PTFE grafts. No particularly significant differences in outcomes were observed based on graft type. Renal function deterioration was observed in 3 patients (3%) in the FCOB group and 4 patients (4%) in the ET group (*p* = 0.140). Myocardial infarction rates were comparable between the groups, occurring in 2 patients (2.4%) in the FCOB group and 3 patients (2.5%) in the ET group (*p* = 1.0).

Restenosis occurred significantly more frequently in the ET group compared to the FCOB group (24 patients, 20.4% vs. 5 patients, 6.1%; *p* = 0.00441). Conversely, reocclusion was significantly more common in the FCOB group than in the ET group (16 patients, 19.5% vs. 8 patients, 6.8%; *p* = 0.00800).

TLR was required in 17 patients (20.7%) in the FCOB group and 24 patients (20.3%) in the ET group (*p* = 0.48). In the FCOB group, anastomotic stenosis occurred in 5 patients (6.1%), of whom 3 were successfully treated endovascularly, while 2 required open graft revision. The remaining 12 patients in this group underwent surgical thrombectomy or graft revision for reocclusion. In the ET group, restenosis of the stent occurred in 24 patients (20.4%). Of these, 18 were managed endovascularly, with 8 treated by angioplasty alone and 10 requiring additional stent placement. The remaining 6 patients underwent surgical revascularization: 3 underwent FCOB, while 2 required unilateral iliofemoral bypass.

TER was performed in 14 patients (17.1%) in the FCOB group and 19 patients (16.1%) in the ET group (*p* = 0.84). In the FCOB group, TER was performed in 5 patients (6.1%) with open thrombectomy due to distal extremity occlusion, while the remaining cases underwent additional endovascular procedures with stent. In the ET group, TER included open thrombectomy in 3 patients (2.5%) and endovascular thrombectomy in 2 patients (1.7%), whereas the remaining cases required additional distal endovascular interventions with stent to restore perfusion.

## 4. Discussion

This retrospective study found no significant differences in primary patency, primary assisted patency, secondary patency, or limb salvage rates between the FCOB and ET groups. However, significant differences were observed in postoperative hospital stay (*p* < 0.0001) and survival rates in the first year (*p* = 0.039), favoring ET. While both techniques are widely utilized, evidence comparing their outcomes for iliac occlusive disease remains limited. This highlights a gap in the literature and underlines the importance of the current study providing a detailed comparison of these two approaches.

In recent years, there has been a clear shift toward ET as the primary method for treating complex iliac occlusive lesions, due to its advantages, such as reduced perioperative morbidity, lower mortality, and shorter hospital stays. Most patients were treated with ET, which has gained popularity due to advancements in techniques like self-expanding covered stents. These advancements have shown good long-term results, even for difficult TASC II C and D lesions [16]. However, surgical options like FCOB continue to play a critical role, particularly in cases involving long iliac occlusions where endovascular techniques may provide suboptimal long-term outcomes [17].

Both TASC II and GLASS classifications showed no significant differences between the FCOB and ET groups in terms of lesion complexity. Similarly, distal runoff patterns were comparable between the groups, further confirming balanced baseline anatomical and clinical characteristics, allowing for an unbiased comparison of the two treatment modalities. The median lesion length was 5.3 cm in the FCOB group and 4.7 cm in the ET group, with no significant difference, but highlighting the preference for FCOB in more extensive and anatomically challenging lesions. This observation aligns with findings from Aburahma et al., who demonstrated that lesion lengths exceeding 5 cm significantly impact the success of revascularization procedures, particularly in maintaining long-term patency [18].

Post-revascularization ABI values showed significant improvement, increasing from 0.45 in the FCOB group and 0.51 in the ET group pre-intervention (*p* = 0.15) to 0.77 and 0.81 post-intervention, respectively (*p* = 0.40). These results confirm the effectiveness of both methods in restoring hemodynamic function, with ABI consistently highlighted in previous studies as a key indicator of procedural success [5,18,19].

The results of this study demonstrate substantial clinical improvements in both groups, as evidenced by the changes in Rutherford categories. Patients undergoing FCOB bypass showed a slightly better improvement in Rutherford categories compared to those treated with ET, though the difference was not statistically significant. These findings highlight the effectiveness of both approaches in managing symptomatic iliac occlusive disease. The significant reduction in severe ischemia (Rutherford categories 4–6) and the high proportion of patients achieving Rutherford categories 0–2 after revascularization are consistent with previous studies. For example, Papakostas et al. demonstrated that effective revascularization leads to a shift toward lower Rutherford categories, reflecting improved perfusion and symptom relief [20]. Similarly, our study reinforces the importance of revascularization in improving patient-reported outcomes, including reduced pain and improved mobility.

The slightly higher proportion of patients achieving Rutherford category 0–2 in the FCOB bypass group suggests a potential advantage of surgical intervention in patients with more extensive or anatomically challenging lesions. This is consistent with findings from studies such as Elkasaby et al., which emphasized the durability and completeness of open surgical procedures for advanced disease [21].

Major amputations were more frequent in the FCOB group but did not reach statistical significance, mirroring similar trends for minor amputations. Limb salvage rates, while high in both groups, slightly favored ET over the long term. These findings are consistent with prior studies, such as Zhang et al. [5], who reported low limb loss rates across both surgical and endovascular approaches, and Ricco et al. [19] who emphasized strong limb salvage outcomes in bypass groups.

Patency outcomes demonstrated similar trends, with both primary and secondary patency rates being favorable across groups. While ET showed slight advantages in primary patency during the first year, FCOB maintained stable results during extended follow-up. Secondary patency rates for both methods reflected the durability of revascularization techniques, aligning with prior research by Myaima et al. [4] and Müller et al. [22] who highlighted the effectiveness of both surgical and endovascular approaches in complex iliac lesions.

Although both FCOB and ET are widely utilized approaches for managing iliac artery occlusions, studies directly comparing the two techniques are exceedingly rare. The only notable investigation to date, conducted by Whatling et al. [11], primarily focused on short-term outcomes such as procedural patency, complication rates, and costs rather than long-term results. Their study reported a patency rate of 100% at six months for FCOB compared to 52% for ET, underscoring the durability of surgical revascularization. However, the absence of long-term follow-up data limits the ability to fully assess the sustained benefits of these approaches.

In contrast, our study extends the understanding of these techniques by offering detailed comparisons of long-term outcomes, including primary, primary assisted, and secondary patency rates, as well as survival and limb salvage over a more extended follow-up period. Our findings demonstrated that, while both methods are effective for revascularization, FCOB is particularly advantageous in complex or extensive occlusions, aligning with the durable outcomes noted by Whatling et al. [11] Furthermore, our data reveal that ET provides a shorter hospital stay and fewer perioperative complications, confirming its role as a less invasive alternative, especially in patients with significant comorbidities.

Survival outcomes favored ET, particularly during the first year, where differences were statistically significant (*p* = 0.039). This trend may reflect the higher prevalence of comorbidities, such as malignancies and prior coronary artery bypass grafting, in the FCOB group. Despite this, both approaches demonstrated favorable long-term survival rates. Comparisons with prior studies, such as those by Nazzal et al. [23] and Massoni et al. [24], underline the consistency of these findings, with survival rates for ET aligning closely with reported outcomes for the endovascular treatment of iliac artery lesions. These differences in survival outcomes likely highlight variations in patient selection and baseline characteristics rather than intrinsic procedural limitations.

It has shown that FCOB serves as a valuable alternative to anatomic bypass for patients with more complex iliac artery lesions, particularly in those who are not candidates for endovascular treatment or who have extensive lesions unsuitable for ET. Additionally, FCOB is often preferred for patients with poor general health or challenging local conditions, such as a hostile abdomen or significant comorbidities [18]. The study by Ricco et al. highlighted that FCOB is particularly effective in patients without significant superficial femoral artery involvement, further emphasizing the importance of careful anatomical evaluation in treatment planning [19]. Elkasaby et al. reported on the advantages of FCOB in complex iliac occlusions, particularly in cases where endovascular techniques were less favorable due to extensive calcifications or lesion complexity [21]. On the other hand, Powell et al. emphasized the increasing impact of endovascular techniques, citing advancements in stent design and procedural safety, which have significantly improved outcomes for TASC II C and D lesions [25]. Their findings are in line with the observed trend toward shorter hospital stays and reduced perioperative morbidity in ET patients.

The current study demonstrated that ET was associated with shorter hospital stays and fewer perioperative risks, while FCOB exhibited significantly lower restenosis rates (6.1% vs. 20.39%, *p* = 0.00441). However, reocclusion rates were higher in the FCOB group (19.5% vs. 6.8%, *p* = 0.00800). Despite these differences, target lesion reintervention (TLR) and target extremity revascularization (TER) rates did not significantly differ between the two groups (*p* = 0.48 and *p* = 0.84, respectively), confirming the effectiveness of both approaches in maintaining arterial flow.

The difference in restenosis and reocclusion rates between FCOB and ET reflects the distinct failure mechanisms of these approaches. Restenosis following ET is primarily driven by neointimal hyperplasia and stent-related factors, which are more pronounced in smaller-caliber vessels or those with significant calcification [6,22]. On the other hand, reocclusion after FCOB is often associated with graft thrombosis, which can be influenced by altered hemodynamics, or progressive disease in the donor iliac artery and compromised SFA. [19] These findings emphasize the need for individualized treatment selection, considering the balance between long-term patency and the risk of early or late graft/stent failure.

Further, the results of the underlying study confirmed that wound infections and bleeding-related complications were more common in the FCOB group, consistent with previous studies [11,23]. Endovascular techniques, as noted by Müller et al. [22] and Jongkind et al. [26], demonstrated lower complication rates, particularly for access-related issues, which were rarely severe. Higher transfusion rates in the FCOB group were favorably in line with the findings from Jongkind et al. [26], reflecting the increased perioperative risks of open procedures. However, the absence of significant differences in myocardial infarction and amputation rates underscores the effectiveness of both approaches in minimizing severe adverse outcomes.

This study has several limitations. As a retrospective analysis, selection bias cannot be entirely ruled out, as treatment decisions were made based on anatomical and clinical considerations rather than randomization. Additionally, the variability in surgical and endovascular techniques across the study period could have introduced variability in outcomes.

## 5. Conclusions

Femorofemoral crossover bypass (FCOB) and endovascular treatment (ET) both represent effective strategies for managing iliac artery occlusions, demonstrating comparable long-term outcomes in terms of limb salvage and patency rates. ET, as the preferred first-line option, offers advantages in recovery time, shorter hospital stays, and lower complication rates. FCOB remains crucial for complex or extensive lesions. However, the appropriate treatment selection has to be tailored according to both the individual patient’s condition as well as anatomical factors.

## Figures and Tables

**Table 1:** Patient characteristics, risk factors, and comorbidities.

	FCOB (*n* = 82)	ET (*n* = 118)	*p*-Value
Age (mean)	69	66	
Male/Female	59(72%)/23(28%)	86(73%)/32(27%)	
Arterial hypertension	76 (92.7%)	101 (85.6%)	0.17
Dyslipidemia	57 (69.6%)	78 (66.1%)	0.64
Diabetes mellitus	16 (19.5%)	30 (25.4%)	0.39
CAOD	29 (35.4%)	45 (38.1)	0.76
Stroke	10 (12.2%)	11 (9.3%)	0.63
Dialysis	1 (1.2%)	2 (1.7%)	1.0
Coronary heart disease	28 (34%)	39 (33%)	0.88
ACBP	13 (15.9%)	6 (5.1)	0.01
Atrial fibrillation	10 (12.2%)	13 (11%)	0.82
Current smoker	35 (42.7%)	80 (67.8%)	0.00049
Former smoker	27 (32.9%)	27 (22.9%)	0.14
ASA 1	0 (0%)	1 (0.85)	1.0
ASA 2	15 (18.3)	28 (23.7%)	0.38
ASA 3	65 (79.3%)	85 (72%)	0.31
ASA 4	2 (2.4%)	3 (2.5%)	1.0
Claudication	37 (45%)	55 (46%)	0.88
CTLI	45 (54.9%)	63 (53.4%)	0.88

Abbreviations: FCOB = femorofemoral crossover bypass, ET = endovascular treatment, CAOD = cerebral arterial occlusive disease, ACBP = aortocoronary bypass grafting, ASA = American Society of Anesthesiologists classification, CTLI = chronic limb-threatening ischemia.

**Table 2:** Lesion classification and anatomical characteristics.

	FCOB (*n* = 82)	ET (*n* = 118)	*p*-Value
Classification and Anatomy			
TASC II C	43 (52.4%)	68 (57.6%)	0.47
TASC II D	39 (47.6%)	50 (42.4%)	0.47
GLASS 1	56 (68.3%)	79 (66.9%)	0.85
GLASS 2	15 (18.3%)	21 (17.8%)	0.92
GLASS 3	11 (13.4%)	18 (15.3%)	0.72
Distal Runoff 1	18 (21.9%)	22 (18.6%)	0.59
Distal Runoff 2	39 (47.6%)	56 (47.5%)	1.0
Distal Runoff 3	25 (30.5%)	40 (33.9%)	0.64

Abbreviations: FCOB = femorofemoral crossover bypass; ET = endovascular treatment; TASC II = Trans-Atlantic Inter-Society Consensus II classification system for categorizing aortoiliac occlusive disease based on lesion complexity; GLASS = Global Limb Anatomic Staging System classification used to stage femoropopliteal and infrapopliteal lesions, guiding revascularization strategies; Distal runoff = grading of distal arterial circulation quality, assessing the number and patency of tibial arteries.

**Table 3:** Postoperative complications and clinical outcomes.

	FCOB (*n* = 82)	ET (*n* = 118)	*p*-Value
Local Complications			
Superficial SSI	12 (14.6%)		
Deep SSI	7 (8.5%)		
Bleeding/Pseudoaneurysm	5 (6.1%)	7 (5.9%)	1.0
Distal Embolization	5 (6.1%)	5 (4.2%)	0.74
Vessel Perforation	0 (0%)	1 (0.8%)	0.31
Systemic Complications			
Anemia/Blood Transfusion	11 (13.4%)	4 (3.4%)	0.01226
Myocardial Infarction	2 (2.4%)	3 (2.5%)	1.0
Renal Function Deterioration	3 (3.7%)	4 (3.4%)	0.92
Clinical Outcomes			
Major Amputation	5 (6.1%)	2 (1.7%)	0.12
Minor Amputation	3 (3.7%)	2 (1.7%)	0.40
Restenosis	5 (6.1%)	24 (20.4%)	0.00441
Reocclusion	16 (19.5%)	8 (6.8%)	0.00800
Target Lesion Reintervention	17 (20.7%)	24 (20.3%)	0.48
Target Extremity Revascularization	14 (17.1%)	19 (16.1%)	0.84

Abbreviations: FCOB = femorofemoral crossover bypass; ET = endovascular treatment; SSI = surgical site infection (superficial or deep postoperative wound infection); Distal embolization = occlusion of small arteries downstream due to embolic debris from the treated lesion; Restenosis = recurrence of narrowing within a previously treated arterial segment; Reocclusion = complete blockage of a previously treated arterial segment; Target lesion reintervention (TLR) = repeat intervention at the originally treated lesion; Target extremity revascularization (TER) = repeat revascularization in the treated limb.

## Data Availability

The data underlying this article will be shared on reasonable request to the corresponding author.
